# 

**Published:** 1895-07-13

**Authors:** 


### CONTENTS.

The Misuse of Hospitals and Honorary Medical Service - 247

The British Medical Association and Antiseptic Surgery - 248

Chloroform Fatalities.—II. By Sir BENJAMIN WARD RICHARDSON, M.D., F.R.S. - 249

Modern Sociology.—Concerning Old Age Pensions - 250

Annotations.—Are Scientific Giants Extinct?—A New Lung for St. George the Martyr—Charity and Brains - 251

Notes and News - 252

Scraps and Gleanings - 261

Medical Progress and Hospital Clinics—

The Treatment of Pott's Disease - 253

PROGRESS IN MEDICINE.—Diseases of the Respiratory System - 255

PROGRESS IN SURGERY.—Cerebral Surgery (*continued*) - 256

NEW APPLIANCES AND THINGS MEDICAL - 258

The Institutional Workshop—

EPSOM COLLEGE - 259

THE WESTMINSTER HOSPITAL - 259

HOSPITAL FESTIVALS AND MEETINGS - 260

The Book World of Medicine and Science - 261

The Student of Medicine - 261

EXTRA SUPPLEMENT.—THE NURSING MIRROR.

News from the Nursing World.—"The Hospital" Convalescent Fund—School of Medicine for Women—The London Hospital—"Her Majesty's Hospital," Stepney—Good Bandaging Appreciated — Leisure in the Workhouse — Ladies' Work at Exeter—Third and Fourth Thousand—Long Hours—Still Without Night Nurses—Queen's Nurses Appreciated Nursing (?) in an Irish Workhouse — Women's Work in Paris — Short Items - xcvii

Elementary Anatomy and Surgery for Nurses. By W. MCADAM ECCLES, M.D., M.S., F.R.C.S. XXV.—The Nervous System (*continued*) - xcix

Hints on the Private Nursing of Mental Cases - c

Everybody's Opinion - ci

Novelties for Nurses - ci

Presentations - ci

Care of the Sick in Alexandria and Cairo. III.—The Greek Hospital - ci

Minor Appointments - ci

Wants and Workers - ci

Holidays and Health - cii

Royal British Nurses' Association - cii

Notes and Queries - cii

